# Immunomodulating effects of the anti-viral agent Silibinin in liver transplant patients with HCV recurrence

**DOI:** 10.1186/s13737-016-0030-7

**Published:** 2016-01-20

**Authors:** Antonino Castellaneta, Antonio Massaro, Maria Rendina, Francesca D’Errico, Sonia Carparelli, Salvatore Fabio Rizzi, Angus W. Thomson, Alfredo Di Leo

**Affiliations:** Department of Emergency and Organ Transplantation, Unit of Gastroenterology, University Hospital, University of Bari, Piazza G. Cesare 11, 70124 Bari, Italy; Department of Surgery, Thomas E. Starzl Transplantation Institute, University of Pittsburgh School of Medicine, 200 Lothrop Street, Pittsburgh, PA 15261 USA

**Keywords:** Silibinin, Liver transplantation, HCV recurrence, Dendritic cells, Regulatory T cells

## Abstract

**Background:**

Silibinin has been shown to have anti-HCV activity and immune-modulating properties by regulating dendritic cell (DC) function. DCs are antigen-presenting cells that, together with regulatory T cells (Treg), play a pivotal role in controlling alloimmune, as well as anti-HCV immune responses.

**Methods:**

Twelve liver transplant patients with HCV recurrence received iv infusion of Silibinin (iv-SIL) for 14 consecutive days. Using flow cytometry, before and at the end of treatment, we determined the frequencies of circulating myeloid (m) and plasmacytoid (p) DC and Treg and the expression of costimulatory/coregulatory molecules by the DC subsets and Treg. Statistical analysis was performed using the paired Student’s *t* test and Pearson correlation test.

**Results:**

After iv-SIL treatment, we observed an elevated plasmacytoid dendritic cell (pDC)/myeloid dendritic cell (mDC) ratio, while pDC displayed lower HLA-DR and higher immunoglobulin-like transcript 4 (ILT4), CD39, and HLA-G expression compared to the pretreatment baseline. In addition, after iv-SIL, mDC showed increased inducible costimulator ligand (ICOSL) expression. No changes were detected in Treg frequency or programed death (PD)-1 expression by these cells. Moreover, several correlations between DC/Treg markers and clinical parameters were detected.

**Conclusions:**

This descriptive study, in liver transplant patients with HCV recurrence, reveals the impact of iv-SIL on DC and Treg. The changes observed in circulating pDC and mDC that have previously been associated with tolerogenic conditions shed new light on how iv-SIL may regulate anti-viral and alloimmunity. We have also observed multiple clinical correlations that could improve the clinical management of liver transplant patients and that deserve further analysis.

## Background

Recently, several experimental and clinical studies have described the anti-oxidative, anti-fibrotic and anti-viral properties of Silibinin, a flavonolignan representing the main component (60 %) of silymarin extracted from the milk thistle (*Silybum marianum gaertneri*). In vivo and in vitro studies have shown that the anti-HCV effect of Silibinin involves stimulation of toll-like receptor (TLR) 7, interferon regulatory factor 3, and p38 mitogen-activated protein kinase pathways [1]. The safety and efficacy of Silibinin as anti-viral therapy has been demonstrated in chronic HCV-infected patients and in liver transplant patients with HCV recurrence unresponsive to standard combined therapy with interferon/ribavirin [2].

There is evidence that Silibinin has also immune-modulating properties and polarizes Th1/Th2 immune responses through functional modifications of dendritic cells (DCs) [3]. DCs are innate immune cells that are also important in the induction and regulation of adaptive immunity. Several subsets of DC have been identified, principally conventional myeloid (m) DC and also type-1 interferon (IFN)-producing plasmacytoid (p) DC, that may be specialized for modifying the quality, strength, and duration of immune responses [4, 5].

Silibinin has been shown to suppress the expression of MHC class I and II and costimulatory molecules (CD80, CD86) by murine bone marrow-derived DC, and this phenomenon has been associated with impairment of LPS-induced IL-12 secretion by these cells [3]. Moreover, Silibinin-treated DCs are highly proficient at antigen capture mediated via mannose receptor-mediated endocytosis and fail to induce Th1 responses and a normal cell-mediated immunity [3].

The outcome of HCV-related hepatitis depends on the balance between immune reactivity and immune suppression/tolerance. After viral infection, activated DCs induce the differentiation of naïve T cells into virus-specific CD4^+^ (IFN-γ; anti-viral Ab) and CD8^+^ T cells for adaptive immunity. It appears that via TLR7 plasmacytoid dendritic cell (pDC) sense HCV-RNA that is transfered inside the pDC after their contact with infected cells, thus inducing type-I IFN secretion. Despite these events, with potential to control and eliminate viral infection, the immune response fails to eliminate the virus in most HCV infections. During the past few years, several authors have demonstrated functional impairment of DC in HCV-infected patients. Thus, Nattermann et al. [6] have shown decreased numbers of BDCA1^+^ (myeloid dendritic cells (mDC)) and BDCA2^+^ DC (pDC) in peripheral blood of HCV-infected patients, together with a concomitant increase in their numbers in liver tissue. The authors suggested that the interaction of the HCV E2 Ag with CD81, a member of the tetraspanin family (considered the cellular receptor for entry of HCV expressed on DC), could result in impaired migration of DC towards the chemokine CCL21 that modulates DC trafficking from sites of infection to lymphoid tissue, with consequent impairment of T cell priming. In addition, decreased CD86 expression and IL-12 secretion by DC from chronically HCV-infected patients has been detected, along with impaired allogeneic T cell stimulatory activity of mDC and reduced IFN-γ and increased IL-10 secretion by T cells. It has been reported that the functional impairment of DC in HCV-infected patients may be related to the presence of HCV-RNA in both mDC and pDC [7]. By contrast, several observations show a normal ability of DC from chronically HCV-infected patients to prime T cells suggesting the need for improved understanding of the role of DC subsets in HCV pathogenesis [8–10].

Recently, inhibition of effector T cells by regulatory T cells (Treg) and the PD-1/PD-L1 pathway has been described as a mechanism responsible for regulating adaptive T cell responses to HCV [11]. Treg are defined as a subset of CD4^+^T lymphocytes that highly express CD25 (IL-2 receptor α-chain) on their surface and the transcription factor Foxp3 intracellularly and constitute approximately 5 % of peripheral CD4^+^ T lymphocytes. Treg critically suppress HCV-specific lymphocyte proliferation, differentiation and cytokine production [11] and promote alloimmune tolerance [12–15].

Regulation of T cell responses, including the induction of Treg, has been shown to be under the control of immune regulatory molecules expressed by DC subsets, including ICOSL (inducible costimulator ligand), PD-L1 (B7 homologue-1 = programmed death ligand-1; CD274), CD39 (ectonucleoside triphosphate diphosphohydrolase-1), and the non-classical HLA class I molecule HLA-G and its receptor, immunoglobulin-like transcript 4 (ILT4) [12]. However, enumeration of DC subsets and Treg and the expression of these immune regulatory molecules on DC subsets and PD-1 on Treg have not been examined in relation to HCV recurrence and Silibinin treatment after liver transplantation.

In this study, we examined peripheral blood DC subsets, Treg, and the expression by these cells of immune regulatory molecules in stable liver transplant patients with HCV recurrence treated for 14 consecutive days with iv infusion of Silibinin (iv-SIL). Our findings reveal changes in circulating pDC and mDC that have previously been associated with tolerogenic conditions (elevated pDC/mDC ratio, increased expression of ILT4, CD39, and HLA-G on pDC and increased expression by mDC of ICOSL) suggesting how iv-SIL might regulate anti-viral and alloimmunity. We have also observed multiple clinical correlations that could improve the clinical management of liver transplant patients and that deserve further analysis.

## Methods

### Study population and treatment

Twelve, clinically-stable, adult liver transplant recipients with normal graft function were eligible for the study (Table 1) and received iv-SIL (20 mg/kg/day) for 14 consecutive days. Eligible patients were males or females aged ≥18 and ≤70, with a ≥1 year posttransplant follow-up, positive serum HCV-RNA by PCR, presence of liver fibrosis as assessed by liver biopsy and/or Fibroscan, with absence of biochemical, clinical, and/or histological evidence of rejection. Female patients of child-bearing age agreed to use a contraceptive method and had a negative pregnancy test at screening. Exclusion criteria included active hepatocellular carcinoma or other neoplasia (excluding cutaneous carcinoma), active biliary tract anomalies, a rejection episode in the 6 months preceding study inclusion, active interferon treatment, creatinine clearance <50 ml/min, history of drug, alcohol or other substance abuse, or other factors limiting their ability to co-operate during the study.

**Table 1:** Demographics of study population

No. patients		12
Age at transplant, yr ± SD (range)		52.88 ± 4.70 (44–60)
Time posttransplant, yr ± SD (range)		7.70 ± 6.31 (1–21)
Age at study, yr ± SD (range)		60.58 ± 7.10 (48–70)
Gender (M:F)		10:2
Immunosuppression at study		
	Tacrolimus	7/12
	Cyclosporine	2/12
	mTor inhibitors	3/12
ALT, U/L ± SD		131.4 ± 117.2
AST, U/L ± SD		85.0 ± 52.6
Total bilirubin, mg/dl ± SD		1.79 ± 2.31
HCV genotype (1/2/3; %)		75/8/17
IL28B genotype (CC/CT/TT; %)		25/50/25
HCV-RNA (log 10 IU/ml; mean ± SD)		6.38 ± 0.58

This study was reviewed and approved by the Ethics Committee of the Ospedale Policlinico–Università degli Studi di Bari, Italy, and patients gave their written informed consent to be part of the study.

### HCV-RNA level, HCV genotype, and IL-28B polymorphism

HCV-RNA levels and HCV genotype were determined using the VERSANT^®^ HCV RNA 1.0 Assay (kPCR) and VERSANT^®^ HCV genotype 2.0 Assay (LIPA), respectively, according to the manufacturer’s instruction (Siemens, Munich, Germany).

DNA for IL-28B polymorphism assay was extracted by using the QIAamp DNA Mini Kit (Qiagen, Hilden, Germany) according to manufacturer’s instructions. Real-time PCR was performed on the ABI Prism 7900HT Sequence Detection System (Applied Biosystems, Monza, Italy). Primers and probes for allelic discrimination for homozygotic (CC and TT) and heterozygotic (CT) genes were as follows: Forward 5′GTGCCTGTCGTGTACTGAACCA3′, Reverse 5′AGCGCGGAGTGCAATTCA3′, Probe_C FAM-CCTGGTTCGCGCCTT-MGB, Probe_T VIC-CCTGGTTCACGCCT-MGB.

### Peripheral blood mononuclear cell isolation and cryopreservation

Peripheral venous blood samples were collected and peripheral blood mononuclear cells (PBMC) isolated, cryopreserved, and recovered, as described in detail [16]. We have shown previously [17] that results obtained staining cryopreserved PBMC do not differ significantly from those obtained using freshly-isolated cells.

### DC subset analysis

As described previously in detail [16], PBMC were stained on melting ice with a lineage (lin) fluorochrome-conjugated monoclonal antibody (mAb) cocktail (anti-CD3, -CD14, -CD19, -CD20). The cells were also stained at the same time with the DC subset-specific mAbs blood DC Ag (BDCA)-1 (CD1; clone AD5-8E7) and BDCA-2 (CD303; clone AC144) (both from MiltenyiBiotec, Auburn, CA), as well as for HLA-G (clone 87G), CD85d (ILT4; clone 42D1) (both from eBioscience, San Diego, CA) HLA-DR (clone L243), CD86 (clone IT2.2), CD83 (clone HB15e), PD-L1 (clone MIH1), CD39 (clone TU66), and the inducible costimulator ligand (ICOSL; clone 2D3/B7-H2) (all from BD PharMingen, San Diego, CA). DC subsets were identified as mDC (lin^−^BDCA-1^+^BDCA-2^−^) or pDC (lin^−^BDCA-1^−^BDCA-2^+^). DC phenotype was further characterized by flow cytometric analysis (FACS-Canto, BD Bioscience), gating on either the mDC or pDC population. Data were analyzed using Facs Diva software (BD Bioscience). Due to low cell recovery for DC analysis in 2 patients, data from 10 instead of 12 patients were available (demographic, clinical, and viral load data were not different).

### Treg analysis

PBMC were stained with fluorochrome-conjugated anti-CD4 (RPA-T4), anti-CD25 (M-A251), anti-CD3 (UCHT1), anti-CD127 (hIL; 7R-M21), anti-PD-1 (MIH4), and anti-Foxp3 (259D/C7) mAbs from BD PharMingen. Intracellular staining for Foxp3 was conducted after surface staining with anti-CD3, -CD4, -CD127, and -CD25 mAbs, as recommended by the manufacturer (BD PharMingen). Treg were defined as CD4^+^CD127^−^CD25^hi^Foxp3^+^ by flow cytometry, and the results expressed as percent total CD4^+^ cells. Foxp3 expression was also expressed as mean fluorescence intensity (MFI).

### Statistical analysis

Results are expressed as arithmetic means ± SD and box and whiskers plots. Statistical analyses were performed using the paired Student’s *t* test and Pearson correlation test. Two-tailed *P* values <0.05 were considered significant.

## Results

### iv-SIL treatment significantly reduces HCV viral load in liver transplant patients

**Fig. 1:**
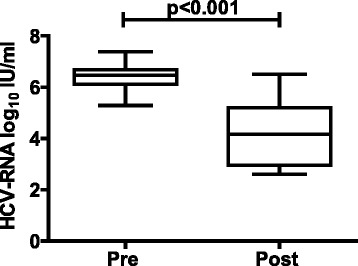
HCV viral load is reduced significantly in liver transplant patients treated with iv-SIL (20 mg/kg/day) for 14 consecutive days. The *box* and *whisker plots* show mean and min to max values before (pre) and after treatment (post)

Twelve liver transplant patients with established HCV recurrence were treated for 14 days with iv-SIL (20 mg/kg/day, i.v.). As previously shown in a larger cohort of patients [2], on day 14 of treatment, HCV viral load (Fig. 1) decreased significantly compared with the pretreatment level (6.38 ± 0.58 vs 4.19 ± 1.25 log_10_ IU/ml). Sixteen days after the end of treatment, viral load mean values were similar to baseline (6.14 ± 0.71 log_10_ IU/ml; data not shown). The treatment was well-tolerated, with no changes in immunosuppressant trough levels and with no dosage adjustments required.

### iv-SIL treatment is associated with an elevated pDC/mDC ratio

**Fig. 2:**
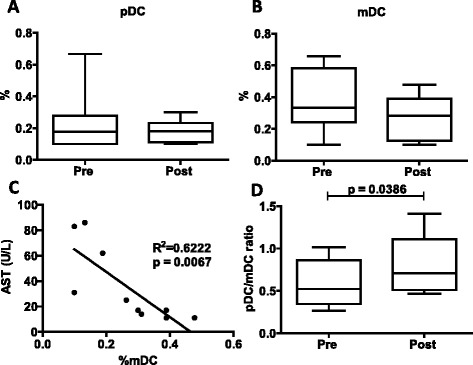
iv-SIL treatment elevates circulating pDC/mDC ratio. PBMC were analyzed before (pre) and after (post) iv-SIL treatment by flow cytometry, as described in the “Methods” section. The frequencies of pDC (**a** Lin^−^BDCA-2^+^) and mDC (**b** Lin^−^BDCA-1^+^), correlation between mDC and AST at the end of treatment (**c**), and the pDC/mDC ratio (**d**) are shown

Peripheral blood DC subset analysis has been shown to be helpful in the immunological monitoring of stable liver transplant patients [12, 17, 18] and those undergoing rejection [19]. In the present study, we analyzed circulating DC subsets by flow cytometric analysis, before and after iv-SIL, as described in the “Methods” section. As shown in Fig. 2, the BDCA-2^+^ pDC frequency was not modified significantly by 14 consecutive days of iv-SIL (Fig. 2a) nor was the frequency of Lin^−^BDCA-1^+^ mDC at the end of iv-SIL treatment (Fig. 2b). However, the mDC frequency at the end of the treatment was inversely correlated with the serum AST level (Fig. 2c). Notably, when the pDC/mDC ratio was calculated, a significantly higher ratio was detected at the end of treatment (Fig. 2d, 0.58 ± 0.27 vs 0.79 ± 0.31, *p* < 0.0386), but no correlation with ALT level, total bilirubin, HCV genotype, or IL-28B polymorphism [20] at baseline or at the end of treatment was observed (data not shown).

### iv-SIL modulation of costimulatory and coregulatory molecule expression by peripheral blood DC subsets

**Fig. 3:**
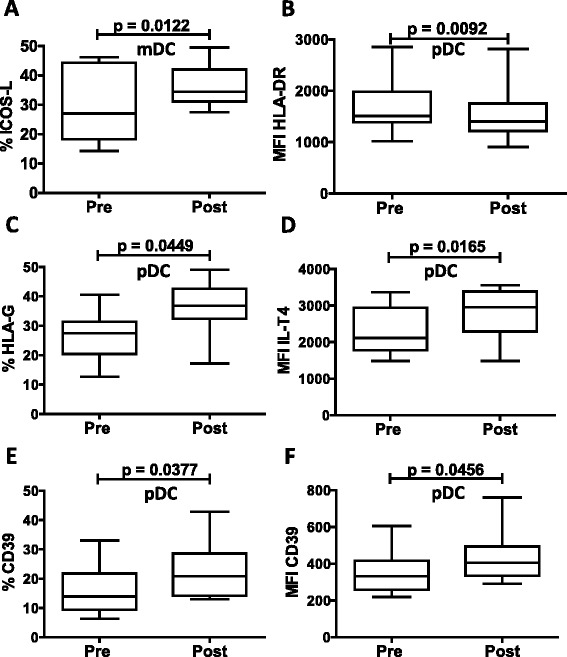
Modulation of costimulatory and coregulatory molecules by iv-SIL. PBMC were analyzed before (pre) and after (post) iv-SIL treatment by flow cytometry, as described in the “Methods” section. Overall analyses of significantly modified markers on mDC (**a** ICOSL) and pDC (**b** HLA-DR MFI, **c** HLA-G %, **d** ILT4 MFI, **e** CD39 %, and **f** CD39 MFI) are shown

DC function and the outcome of DC-T cell interactions may depend on the net costimulatory and coregulatory signals delivered by DC. Thus, we used flow cytometric analysis to identify and quantify selected immune costimulatory/coregulatory molecules (CD83, CD86, ICOSL, PD-L1, HLA-G, ILT4, CD39) and HLA class II on circulating mDC and pDC before and after 14 consecutive days of treatment with iv-SIL. The data (Tables 2 and 3) show that after iv-SIL exposure, the expression of costimulatory and coregulatory molecules on circulating mDC was not modified significantly, except for higher expression of the coregulatory molecule ICOSL (Fig. 3a, %: 29.6 ± 12.6 vs 36.2 ± 7.2; *p* < 0.0122). By contrast, after iv-SIL, pDC exhibited a modest but significant downregulation of HLA-DR expression (Fig. 3b; MFI: 1673.5 ± 525.4 vs 1523.4 ± 531.1; p < 0.0092) and upregulation of the non-classical HLA class I molecule HLA-G (Fig. 3c; %: 26.2 ± 8.1 vs 36.1 ± 8.6; *p* < 0.0449) and its receptor ILT4 (Fig. 3d; MFI: 2303.6 ± 632.8 vs 2743.4 ± 718.6; *p* < 0.0165). Moreover, pDC from iv-SIL patients exhibited higher expression of the ectonucleosidase CD39 (Fig. 3e: 16.2 ± 8.7 vs 22.1 ± 9.4 %; *p* < 0.0377; Fig. 3f: MFI: 349.7 ± 116.9 vs 437.8 ± 143.6; *p* < 0.0456) compared with cells isolated before iv-SIL treatment. No significant change in PD-L1/CD86 ratio was found after iv-SIL (Table 3). Although the magnitude of these differences is small, these findings suggest that by modulating costimulatory and coregulatory molecule expression by peripheral blood DC subsets, iv-SIL could influence the immune response.

**Table 2:** Modulation of costimulatory and coregulatory molecule expression by iv-SIL

% (mean ± SD)	pDC			mDC		
	Pre	Post	*p*	Pre	Post	*p*
HLA-DR	92.9 ± 5.5	90.9 ± 7.8	0.1600	93.7 ± 9.7	87.5 ± 17.3	0.1035
CD83	45.9 ± 16.2	50.3 ± 20.6	0.6231	26.7 ± 10.7	37.7 ± 23.1	0.1793
CD86	59.8 ± 14.2	65.6 ± 10.9	0.2250	90.6 ± 5.3	87.8 ± 14.0	0.6306
ICOSL	45.1 ± 18.3	52.2 ± 12.3	0.2758	29.6 ± 12.6	36.2 ± 7.2	*0.0122*
PD-L1	18.9 ± 11.2	25.5 ± 12.0	0.0883	10.6 ± 6.8	17.0 ± 10.6	0.0938
HLA-G	26.2 ± 8.1	36.19 ± 8.6	*0.0449*	45.8 ± 10.3	50.8 ± 11.6	0.3614
IL-T4	97.3 ± 2.7	96.8 ± 6.1	0.7293	89.3 ± 5.7	91.7 ± 5.6	0.3397
CD39	16.2 ± 8.7	22.1 ± 9.4	*0.0377*	69.4 ± 7.6	74.0 ± 10.7	0.3064

PBMC were analyzed before and after iv-SIL treatment by flow cytometry, as described in the “Methods” section, and the overall analysis of HLA-DR, CD83, CD86, ICOSL, PD-L1, HLA-G, IL-T4, and CD39 expression (%, mean SD) by mDC and pDC is presented. Italicized values denote significance

**Table 3:** Modulation of costimulatory and coregulatory molecule expression by iv-SIL

MFI (mean ± SD)	pDC			mDC		
	Pre	Post	*p*	Pre	Post	*p*
HLA-DR	1673.5 ± 525.4	1523.4 ± 531.1	*0.0092*	2867.8 ± 440.9	2661.0 ± 573.2	0.1507
CD83	548.5 ± 187.7	755.0 ± 683.6	0.3982	374.9 ± 108.1	620.7 ± 392.5	0.0865
CD86	714.7 ± 174.0	743.6 ± 139.0	0.6359	1462.2 ± 322.8	1303.2 ± 328.0	0.4298
ICOSL	554.1 ± 223.8	640.4 ± 209.8	0.5265	498.2 ± 257.8	501.2 ± 133.7	0.9219
PD-L1	316.1 ± 110.7	376.8 ± 111.7	0.1848	272.0 ± 68.9	326.1 ± 88.6	0.1311
HLA-G	356.7 ± 57.8	409.9 ± 62.3	0.0583	567.7 ± 120.5	587.5 ± 108.4	0.6944
IL-T4	2303.6 ± 632.8	2743.4 ± 718.6	*0.0165*	1797.7 ± 424.0	2294.4 ± 570.7	0.0669
CD39	349.7 ± 116.9	437.8 ± 143.6	*0.0456*	1516.8 ± 269.2	1761.7 ± 527.6	0.2601
PD-L1/CD86	0.447 ± 0.110	0.5217 ± 0.197	0.3830	0.189 ± 0.042	0.268 ± 0.116	0.0929

PBMC were analyzed before and after iv-SIL treatment by flow cytometry, as described in the “Methods” section, and the overall analysis of HLA-DR, CD83, CD86, ICOSL, PD-L1, HLA-G, IL-T4, and CD39 expression (MFI, mean SD) as well as the PD-L1/CD86 ratio on mDC and pDC is reported. Italicized values denote significance

### Correlations between peripheral blood DC subset markers and clinical parameters

**Fig. 4:**
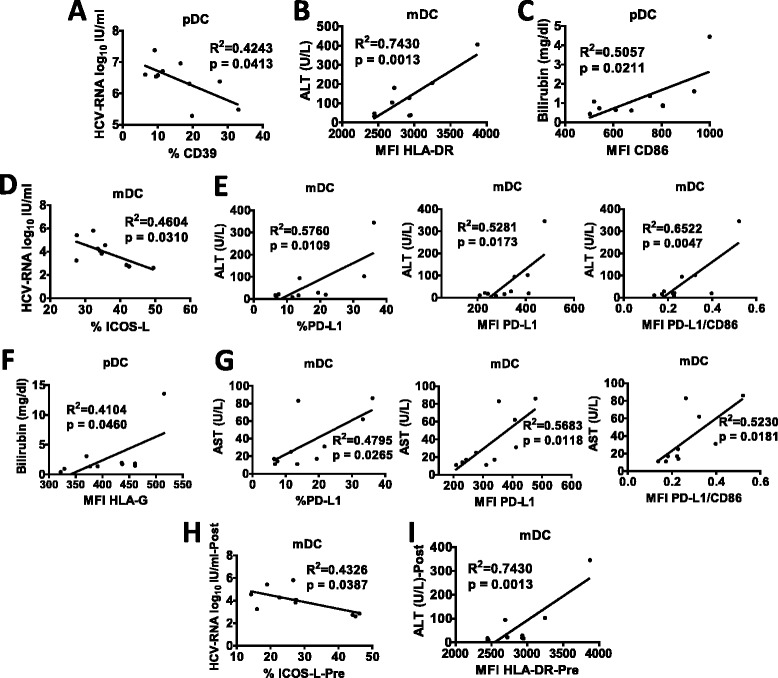
Correlation analyses between DC markers and clinical parameters. AST, ALT, total bilirubin, HCV RNA, HCV genotype, and IL-28B genotype were analyzed in order to verify possible correlations with circulating DC subsets markers before (Fig. 4**a**–**c**) and after iv-SIL treatment (Fig. 4**d**–**g**). DC markers at baseline were also correlated with clinical parameters at the end of iv-SIL treatment (Fig. 4**h**–**i**)

We next analyzed possible correlations between circulating DC subset markers and clinical parameters (AST, ALT, total bilirubin, HCV-RNA, HCV genotype, IL-28B genotype) before (Fig. 4a–c) and after iv-SIL treatment (Fig. 4d–g). In addition, DC markers at baseline were also correlated with clinical parameters at the end of iv-SIL treatment (Fig. 4h–i).

At pretreatment baseline, serum HCV-RNA was inversely correlated with CD39 (%) on pDC (Fig. 4a) and serum ALT was positively correlated with HLA-DR (MFI) on mDC (Fig. 4b), whereas a positive correlation between CD86 expression (MFI) on blood pDC with total bilirubin level was found (Fig. 4c).

At the end of the treatment, there was an inverse correlation between HCV-RNA and ICOSL expression (%) on mDC (Fig. 4d). Moreover, there was a positive correlation between serum ALT and AST and PD-L1 expression (% and MFI) and PD-L1/CD86 ratio on mDC (Fig. 4e, g) and also a positive correlation between HLA-G expression (MFI) on blood pDC and total bilirubin level (Fig. 4f).

Notably, HCV-RNA at the end of treatment was correlated inversely with ICOSL expression on mDC at baseline (Fig. 4h), and serum ALT was positively correlated with HLA-DR expression (MFI) on mDC at baseline (Fig. 4i).

At the moment, data provided by this study do not allow to conclude on the real clinical impact of such correlations. Thus, larger prospective study is needed to validate their clinical significance.

### iv-SILtreatment does not significantly influence the Treg compartment

**Fig. 5:**
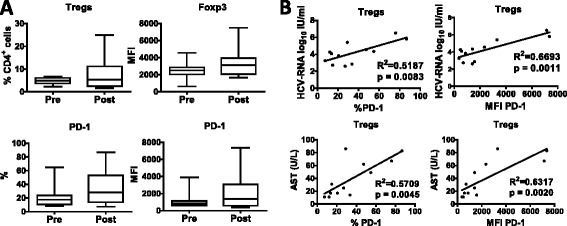
iv-SIL does not influence the Treg compartment. Peripheral blood Treg and their expression of PD-1 were analyzed before (pre) and after (post) iv-SIL treatment by flow cytometry, as described in the “Methods” section. PBMC were stained using a combination of anti-CD3, anti-CD4, anti-CD127, anti-CD25, and anti-Foxp3, and Treg defined as CD4^+^CD127^−^CD25^hi^Foxp3^+^. Almost all CD4^+^CD127^−^CD25^hi^ cells (94 %) expressed Foxp3. **a** Overall analysis of the frequency of circulating Treg and the overall analysis of the Foxp3 (MFI) and PD-1 (% and MFI) expression by Treg. **b** Correlation between PD-1 expression on Treg (% and MFI) and HCV-RNA and AST

Treg and the PD-1/PD-L1 pathways have been described as mechanisms responsible for balancing of HCV adaptive T cell responses [11], and therefore, Treg frequency and PD-1 expression were analyzed by flow cytometry, before and after 14 days of treatment with iv-SIL. As shown in Fig. 5a, Treg frequency, Foxp3 expression, and PD-1 expression were modestly but not significantly increased after iv-SIL treatment. Notably, at the end of treatment, PD-1 expression on Treg (% and MFI) was correlated positively with HCV viral load and serum AST levels (Fig. 5b), whereas no correlation with ALT, total bilirubin, HCV genotype, or IL-28B polymorphism was observed either at baseline or at the end of treatment (data not shown).

## Discussion

Both DC and Treg are believed to play important roles in the regulation of alloimune responses, host responses to HCV infection [21], and transplant outcome [13, 22]. In this study, we have monitored key immunological parameters previously recognized to be involved in the regulation of anti-viral immunity and tolerance/rejection for the first time in liver transplant patients with HCV recurrence treated for 14 consecutive days with iv-SIL. Recently, in a single-center, prospective, randomized, parallel-group, double-blind, placebo-controlled, phase 2 trial, we have shown that Silibinin monotherapy has significant anti-viral activity in patients with established HCV recurrence in the graft and who are unresponsive to standard therapy. We have also confirmed the safety and tolerability of iv-SIL without interaction with immunosuppressive drugs [2]. In the present study, we show that, associated with a significant decrease in HCV viral load, iv-SIL induces changes in circulating DC that have previously been associated with tolerogenic conditions [12, 23], providing new insight into how iv-SIL may regulate alloimmunity and anti-HCV immunity in liver transplant patients. After iv-SIL treatment, we observed a higher pDC/mDC ratio and pDC exhibited reduced HLA-DR and elevated ILT4, HLA-G, and CD39 expression compared with baseline values. In addition, after iv-SIL administration, blood mDC showed higher ICOSL expression. By contrast, no significant changes were detected in Treg frequency or PD-1 expression. Our study also reveals significant correlations between clinical parameters and DC and Treg markers which could prove helpful, especially if confirmed in larger, prospective studies, in the clinical management of liver transplant patients.

In humans, conventional mDC and “non-conventional” pDC exhibit inherent tolerogenic properties in the healthy steady-state, and each plays an important role in regulating innate and adaptative immunity. There is also evidence that pDC, more than mDC, might be predisposed to induce tolerance [24–27]. We have shown that tolerant and prospective weaning liver transplant patients exhibit a significantly higher incidence of circulating pDC relative to mDC compared with those patients requiring maintenance immunosuppression [17]. Moreover, modulation of the pDC/mDC ratio has been proven to have a biological influence not only in the transplant setting but also in other diseases or conditions, such as chronic obstructive pulmonary disease and pregnancy [28, 29]. The findings of the current study now show that after 14 consecutive days of iv-SIL, the pDC/mDC ratio in peripheral blood is higher than at baseline and it does not correlate with any of clinical parameters evaluated, including the viral load. These data therefore suggest that iv-SIL in liver transplant patients may promote redistribution of peripheral blood DC subsets in favor of pDC. Whether or not this finding might be correlated with a regulatory/tolerogenic effect of iv-SIL will be addressed in further studies. Moreover, blood mDC after iv-SIL were modestly but not significantly reduced; their frequency was inversely correlated with serum AST level at the end of the treatment.

Since DC function and the outcome of DC-T cell interactions may depend on the net costimulatory and coregulatory signals delivered by the DC, we tested the expression of several costimulatory and coregulatory molecules on blood DC subsets. Notably, we found that the mean fluorescence intensity of HLA-II DR on pDC was slightly but significantly downregulated after iv-SIL, suggesting impaired ability of pDC to stimulate T cells and immune reactivity to the same extent as baseline pDC. Moreover, CD86 expression (a maturation marker which was not modified significantly by iv-SIL) on circulating pDC was positively correlated with total bilirubin level at baseline.

HLA-G and its receptor ILT4 are believed to play an important role in regulating both the maturation and function of DC. Thus, interaction between HLA-G and its receptors inhibits DC maturation [30]. Moreover, soluble HLA-G inhibits human DC-triggered allogeneic T cell proliferation [31] and HLA-G-expressing antigen-presenting cells induce CD4^+^ T cell Treg [32]. Although not directly demonstrated in the present study, these findings suggest that elevated HLA-G and ILT4 on peripheral blood pDC may be consistent with the ability of iv-SIL to promote a tolerogenic state. Moreover, expression of HLA-G on pDC after iv-SIL positively correlated with total bilirubin level.

We also observed for the first time that after iv-SIL, pDC, but not conventional mDC, exhibited significantly higher levels of CD39 (% and MFI) than at baseline, which was inversely correlated with viral load. It has been shown that maturation of DC, an important control point in the development of immune responses, can also be modulated by extracellular adenosine triphosphate (ATP) released by DC and also by damaged/necrotic cells. CD39 is an ectoenzyme that degrades ATP to AMP and can therefore modulate DC activation and inhibit their capacity to initiate Th1 responses [33]. In the liver transplant setting, it may temper alloimmunity and reduce the risk of graft injury and rejection [34].

The ICOS-ICOSL signaling pathway is involved in Th2 immune response enhancement and in germinal center formation, somatic hypermutation, and class switch recombination [35–37]. Moreover, Treg are thought to develop as a consequence of signaling through costimulatory molecules [38], and some subsets of Treg appear to depend on ICOS-ICOSL signaling pathway for in vivo and in vitro suppression through IL-10 production [39, 40]. Enhanced ICOSL expression by DC has also been shown to be involved in the pathogenesis of infectious disease [41]. The present study demonstrates upregulation of ICOSL on peripheral blood mDC after iv-SIL which was inversely correlated with viral load. Several markers (HLA-DR, PD-L1, and PD-L1/CD86 ratio) on mDC that were not modified by iv-SIL treatment were positively correlated with transaminase levels.

There is evidence that circulating levels of Treg are elevated in tolerant liver transplant patients compared with non-tolerant patients, or healthy individuals [12, 16, 42, 43]. Moreover, the expression of PD-1 on total Treg as well as Treg subsets (naїve/central memory/effector memory/effector) is significantly higher in HCV-infected patients than in healthy controls. In addition, PD-1/PD-L1 signaling negatively regulates Treg by limiting STAT-5 phosphorylation in patients with chronic HCV infection, and PD-1 overexpression at the site of inflammation is involved in the establishment of a long-lasting inflammatory disease. The present study does not show any significant change in the incidence of peripheral blood Treg or Foxp3 expression in liver transplant patients after iv-SIL. Moreover, no change was also found in PD-1 expression by Treg. These findings suggest that despite a significant decrease in viral load by the end of treatment [2], iv-SIL does not affect a possible mechanism (PD-1/PD-L1 signaling on Treg cells) involved in the persistence of HCV infection. This is consistent with clinical findings showing that 16 days after the end of the treatment, the infection persisted with viral load mean values similar to baseline [2]. Interestingly, although not modified by iv-SIL, the expression of PD-L1 on Treg after treatment correlated positively with HCV viral load and serum AST.

Correlations observed in the present study between DC and Treg markers and liver functional tests, including HCV viral load, have not been described previously, and their biological/clinical significance is unclear. Thus, larger prospective studies are needed to validate their clinical significance. In order to discriminate the specific roles of iv-SIL, HCV viral load and patient immune suppression in affecting circulating DC and Treg, functional and mechanistic in vitro studies need to be undertaken.

## Conclusions

In conclusion, we believe that this is the first study in liver transplant patients with HCV recurrence showing the impact of iv-SIL on circulating DC and Treg. The findings described reveal changes in circulating DC subsets that have been associated previously with regulatory/tolerogenic conditions and suggest how Silibinin might regulate alloimmunity and the anti-viral immune response. Moreover, we have observed multiple clinical correlations that could eventually improve the clinical management of liver transplant patients and that deserve further analysis.

## Abbreviations

DC: dendritic cell
IFN: interferon
iv-SIL: iv infusion of Silibinin
Lin: lineage
mAb: monoclonal antibody
mDC: myeloid dendritic cells
MFI: mean fluorescence intensity
PBMC: peripheral blood mononuclear cells
pDC: plasmacytoid dendritic cells
Treg: regulatory T cells
